# An Optical Method for the In-Vivo Characterization of the Biomechanical Response of the Right Ventricle

**DOI:** 10.1038/s41598-018-25223-z

**Published:** 2018-05-01

**Authors:** A. Soltani, J. Lahti, K. Järvelä, S. Curtze, J. Laurikka, M. Hokka, V.-T. Kuokkala

**Affiliations:** 1 Tampere University of Technology, Laboratory of Materials Science, POB 589, FI33101 Tampere, Finland; 2 Tampere University Hospital Heart Center, POB 2000, FI-33521 Tampere, Finland; 3 University of Tampere, Faculty of Medicine and Life Sciences, POB 100, Tampere, FI-33014 Finland

## Abstract

The intraoperative *in-vivo* mechanical function of the left ventricle has been studied thoroughly using echocardiography in the past. However, due to technical and anatomical issues, the ultrasound technology cannot easily be focused on the right side of the heart during open-heart surgery, and the function of the right ventricle during the intervention remains largely unexplored. We used optical imaging and digital image correlation for the characterization of the right ventricle motion and deformation during open-heart surgery. This work is a pilot study focusing on one patient only with the aim of establishing the framework for long term research. These experiments show that optical imaging and the analysis of the images can be used to obtain similar parameters, and partly at higher accuracy, for describing the mechanical functioning of the heart as the ultrasound technology. This work describes the optical imaging based method to characterize the mechanical response of the heart *in-vivo*, and offers new insight into the mechanical function of the right ventricle.

## Introduction

Open-heart surgery is among the most challenging procedures in the medical field. Successful conduction of the surgery and recovery from it is often closely linked with constant and accurate monitoring of the myocardial mechanics and the haemodynamic parameters throughout the surgery, allowing the surgical team to perform immediate interventions if required. Parameters describing the functionality of the heart are, for instance, expressions describing the motion and deformation of the right ventricle (RV). Any abnormality in these can indicate a severe problem in the heart. During the majority of open-heart procedures, cardiopulmonary bypass (CPB) is mandatory, i.e., the mechanical function of the heart is stopped with cardioplegia in the diastolic state, whilst the surgeons operate on the heart. Meanwhile, the CPB circuit takes care of the circulation and oxygenation of the body. After the cardioplegic arrest, the spontaneous heart functions are returned and before the weaning process from the CPB, the heart must recover functionality. An interesting detail worth noticing is the fact that all RV complications reflect on the motion, deformation, and overall movements of the heart either on a local scale (e.g., infarction^1^) or on a global scale (electrical and mechanical dysfunctions^2^). Longitudinal motion of the RV lateral wall is reduced even after uneventful cardiac surgery despite preserved RV ejection fraction and stroke volume^3^. Therefore, better understanding of changes in RV function is necessary, and this in turn requires reliable and reproducible measures.

Recently, strain and strain rate measurements of the RV using echocardiography and magnetic resonance imaging (MRI) have considerably enhanced the understanding of RV functions^4^. As useful as echocardiography is, it has some shortcomings: its poor intraoperative feasibility for the studies of the right side of the heart (ventricle or atrium), the high skill level required from the operator, and the requirement of an intrusive probe (transesophageal echocardiogram) for data gathering and subsequent analysis, among others. An overview of the clinical difficulties in RV assessment using echocardiography has been presented by Teske *et al*.^5^. However, as the right side of the heart is optically visible during the surgery, it is quite straightforward to obtain optical images of it. Thus, Digital Image Correlation (DIC) can be used for online and real-time analysis of the right side of the heart, initially in an academic framework, but most certainly with future potential also for clinical monitoring. Verification of the DIC technique against data from the established ultrasound is subject of our ongoing studies. Although simultaneous acquisition of ultrasound data from patients is difficult due to the mentioned shortcomings of ultrasound, we believe this is the only feasible control study possible. A comprehensive review of application of DIC in the field of biomechanics is presented in ref^6^. While at its infancy in the medical field, DIC has already been used to study soft/organic tissues in earlier studies. Moreover, the advantage of stereo DIC (2 camera system) in analysis of curved surfaces and out of plane movements can be useful for studying mechanical behavior of biological material which are not typically flat.

Numerous studies cover DIC analysis of biological samples *in-vitro*. These studies use either biological tissue or a material that closely resembles biological tissue. Zhang *et al*.^7^ performed uniaxial tension tests for three different samples: arterial tissues, bovine hoof horn, and cemented hip replacements. The conclusion was that by using a proper speckle pattern, DIC could be used to measure the displacements/strains in biological samples. In a study by Rizzuto *et al*.^8^, a DIC analysis algorithm was developed by the authors for biological samples. After testing the accuracy of the algorithm on samples with known strains, the strains on mouse skin samples with no artificial pattern were measured. The results indicated relative errors of less than ~2%. In another work by the same authors^9^, contractility of engineered skeletal muscle tissues was studied with a single high speed camera DIC setup (2D DIC) with acquisition speeds as high as 500 fps. Here, the errors were even lower (~0.15%), showing the great potential of DIC in monitoring the engineered tissue during the process of maturation and growth. As final examples of studies where artificial materials were used, Moerman *et al*.^10^ performed indentation tests on a silicone gel phantom (see ref.^11^) covered with a random speckle pattern. The 3D surface deformation was recorded using a two-camera DIC system (stereo DIC). They concluded that in combination with finite element modeling, stereo DIC has the potential to characterize both surface and bulk properties of soft tissue materials. In another research work, Genovese *et al*.^12^ performed a similar study with a single camera DIC system using a fringe pattern instead of random speckle patterns. The experiment included a radial fringe pattern on a latex foam sample. The DIC analysis results were confirmed for biological tissue by carrying out a reference experiment on a sample with the same dimension cut from a porcine heart.

DIC has been used successfully to study human tissue as well. In a study by Luyckx *et al*.^13^, a two-camera system was used for stereo analysis of human tendon tissue. The authors cited the difficulties of applying a suitable speckle pattern on the samples, especially in the case of moist sample surfaces. Their proposed solution was to use an inverse speckle pattern (black on white background instead of white on black background). This was possible by dying the samples with dark methylene blue and applying the speckle pattern to the surface. According to the results, stereo DIC provided a highly accurate tool for measuring strains on human tendon tissues. In a more medically focused study, Coudrillier *et al*.^14,15^ used donor human eyes to assess the biomechanical response of the posterior sclera. Using a stereo DIC system and a graphite-patterned sclera, they studied the anisotropy of the human eye and the effects of age and glaucoma on the deformation behavior of the sclera. In one of the first *in*-*vivo* studies on live human tissue, Campo *et al*.^16^ compared the recorded pulse wave velocity (PWV) of the arterial system using DIC and ultrasound. According to their findings, PWV measurements from both methods were more or less similar in values. From this, the authors concluded that apart from PWV recording, DIC could be adapted for further applications in biomechanics.

The examples discussed briefly above establish the potential of DIC for studying the mechanical behavior of biological materials. However, no systematic clinical studies have been carried out on monitoring the functions of the human heart with the technique. In a previous study^17^, the authors have already shown that DIC can provide meaningful data on the deformation of the heart and the potential of DIC in the medical and biomechanics fields. However, the preliminary study was limited by the low contrast pattern of the natural texture of the heart, and because of this, the results and conclusions were limited. The present work is a continuation of this previous preliminary work reported in^17–19^, and the aim of the current paper is to quantify the native biomechanical response of the human heart during cardiac surgery. In this paper, we present a methodology for the characterization of the motion, deformation, and functioning of the right ventricle of the heart. The results presented are based on our recent measurements carried out at the Tampere University Hospital Heart Center (Tampere, Finland). Because of the high volume of data available for each surgery, this paper presents the results for only one patient to demonstrate the capabilities of the new method as a proof of concept. Future research topics and possibilities are discussed at the end of the paper.

## Materials and Methods

**Figure 1:**
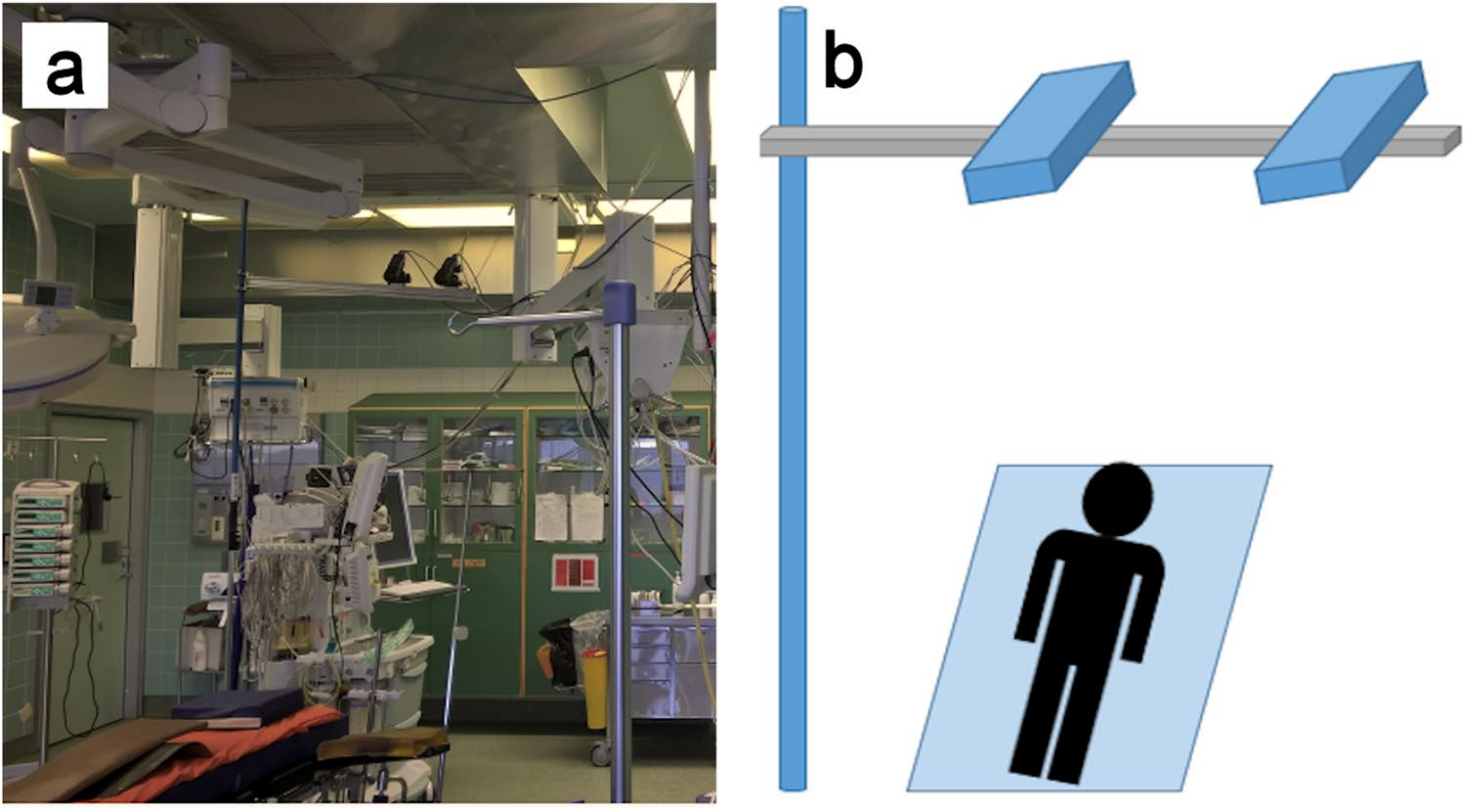
(**a**) Picture of the DIC setup installed in the surgery room, (**b**) Schematic picture of the DIC setup.

The ethical issues and the research protocol regarding the experiments was reviewed and approved by the European Union Drug Regulating Authorities Clinical Trials (2016-000575-24). The selected research methods, documentation, devices, and instruments followed the appropriate regulations governing the clinical research. The study protocol, documentation, as well as the feasibility of all devices and instruments for clinical research were reviewed and approved by the National Supervisory Authority for Welfare and Health (Valvira, Permit no. 330) to verify that the research followed appropriate regulations. The research described in this paper was carried out with full adherence to the approved research protocol and guidelines set by these institutions. Furthermore, an informed written consent was obtained from the patients where they acknowledge receiving the information about the research details and the risks involved in the study. The DIC setup shown in Fig. 1 used two 5-megapixel E-lite cameras equipped with 50 mm Nikon lenses. The equipment was installed in the operating theater using a custom-made vertical rod between the floor and the ceiling with a crossbar carrying the cameras, providing a slanted view of the surgery table. This angled view and the rather long distance between the cameras and the target are non-optimal, but in an operating room, the sterile field directly above the patient is subject to very strict regulations and was therefore not accessible for the cameras. Another challenge with this setup is that the exact distance from the camera lenses to the patient’s chest was not known prior to the surgery, because the position of the patient’s heart varies from surgery to surgery depending on the size of the patient and the preferences of the operating surgeons. Re-adjusting the cameras is not easily possible during the surgery due to the restrictions in interfering with the sterile area. The surgery table was used as a reference point for positioning of the cameras, focusing of the lenses, and calibrating of the stereo system. No further light source was employed in addition to the surgical lights routinely used during the surgery.

**Figure 2:**
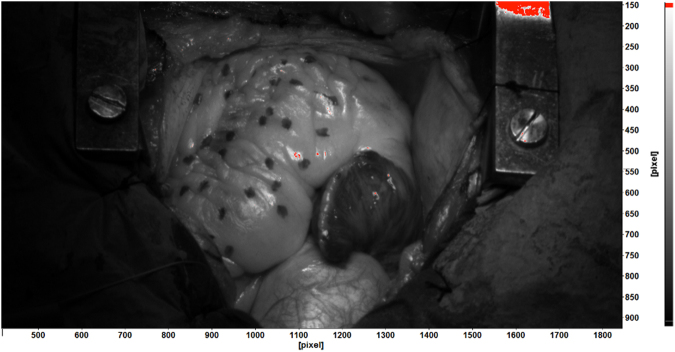
An example of the pattern applied by the surgeons during the surgery using a non-toxic sterile marker.

The surface of the human heart shows very little natural texture, i.e., it has a poor contrast pattern, which makes it poorly suitable for DIC studies. For a large number of patients, a layer of fat covers the outer surface of the myocardium and gives it a uniform white/yellowish appearance. As demonstrated in the previous papers^17–19^, this natural pattern does not provide enough contrast for high resolution DIC. In order to improve the contrast in the present study, the surgeons applied a random dot pattern to the heart’s surface using a non-toxic sterile medical marker. An example of the pattern is shown in Fig. 2.

During the surgical procedure examined in this paper, approximately 200 images were taken at a sampling frequency of ~15–20 Hz after opening the sternum. In this paper, we present the results of one patient to demonstrate the new developed methodology. The detailed analysis of all images is beyond the scope of this paper, and will be dealt with in future publications. The images were analyzed offline after the surgery using the Davis v8.3 software suite provided by LaVision. The analysis took approximately ten minutes per heartbeat. The displacement vectors were obtained using a subset size of 79 pixels and a step size of 10 pixels. A stereo calibration was obtained for each surgery by taking several images of a 3D calibration target. In the calibration results obtained for this surgery, the pixel size was ~0.1 mm and the calibration errors ~0.1 pixels or 0.01 mm. The calibration error in the Davis software is calculated as the root mean square (RMS) difference between the calculated positions of the points in the calibration images and their known positions. The maximum size of the image was 5Mpixels, but the frame was cropped to 2055 by 1788 pixels to increase the frame rate. The sum of differentials routine was used for calculating the displacement vectors. When using this routine, errors accumulate from one image to the next leading to possibly high error values at the end of a long image series. To minimize this, the images for each heartbeat were analyzed separately, and the vector fields of the beats were then “stitched together” as a function of time to cover more than one cardiac cycle.

### Data Availability

The datasets generated during and/or analyzed during the current study are available from the corresponding author on reasonable request.

## Results

In this section, we present two sets of results obtained from the DIC measurements. The first set is exclusive to DIC and includes displacement vector components and principal strains on the surface of the heart. These values are not typically used for diagnostic purposes in the medical field, but they describe the full field motion and biomechanical response of the heart quantitatively. The second set of data includes tissue velocity, strain rate, strain, and fractional shortening^20–22^. These values are important for our study in terms of comparison to results obtained by other techniques, since their usage has already been established for monitoring of the functions of the heart.

**Figure 3:**
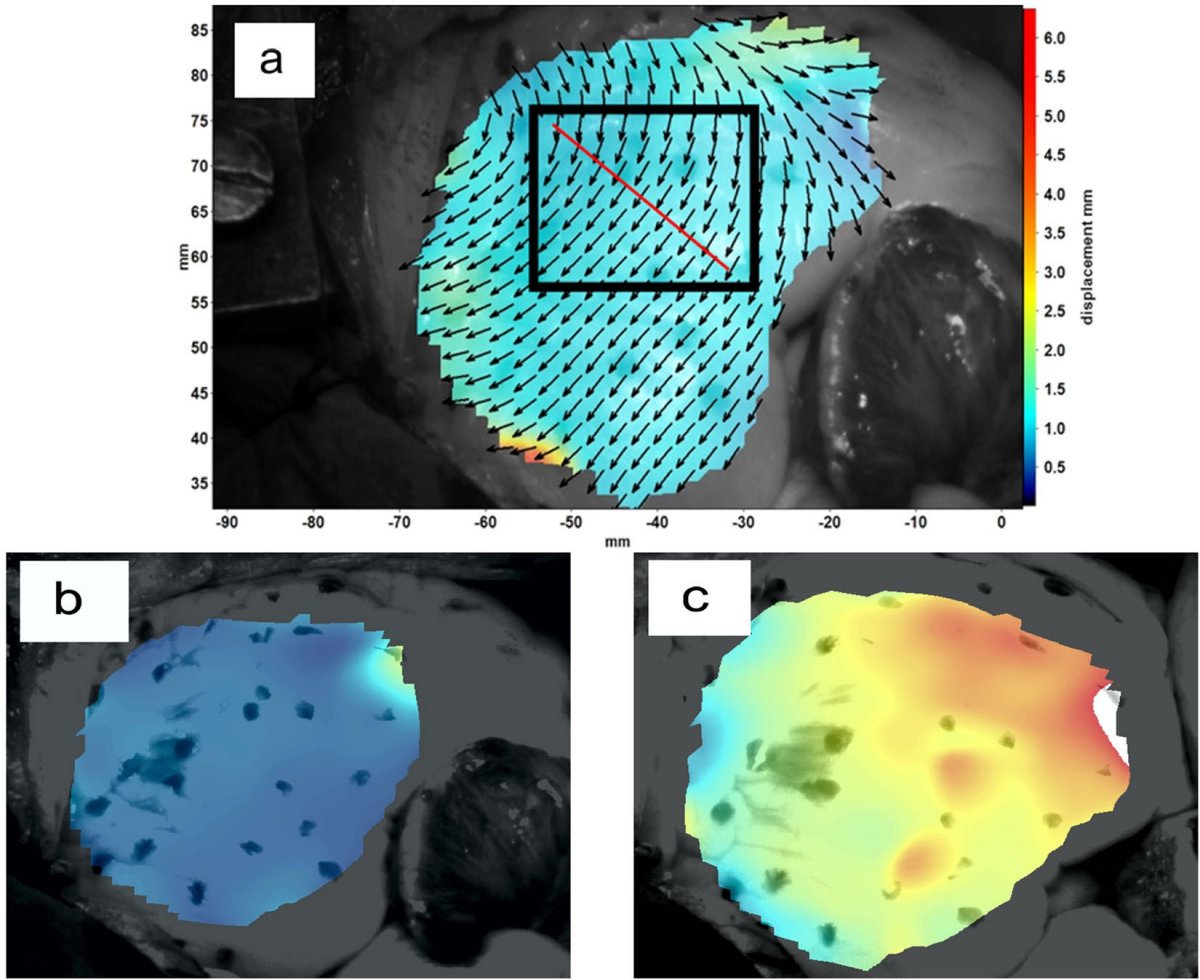
(**a**) Original image from the heart overlain with displacement vectors and the vector length indicated by the colors. The black rectangle indicates where the average of the vector components, strain rate, principal strains, and tissue velocity were obtained from, and the red line shows the direction and length of the virtual extensometer used for extracting the data for fractional shortening (**b**) Displacement color heat maps for early systole stage (**c**) Displacement color heat maps for end diastole stage.

The region of interest used in this paper to calculate the average vector components, average strain rate, and the average principle strains is shown in Fig. 3a. This region was selected such that it covers as much of the RV surface as possible. In addition, the elongation and contraction of the heart or the Lagrangian strain is calculated by using a virtual extensometer across the target surface. The extensometer line that was used is shown in Fig. 3a as well. The X- and Y-axes are by definition aligned horizontally and vertically with the image edges, respectively. Figures 3b,c show displacement color heat maps of the heart’s surface for early systole and end diastole stage, respectively. It should be noted that Fig. 3a does not show all displacement vectors for better readability of the figure. The step size of 10 pixels used in this study produces a vector for every 10th pixel. The background color heat map is a continuous average of the length of the displacement vectors.

In conventional heart monitoring, values such as strain, strain rate, and tissue velocity are obtained using Tissue Doppler Imaging (TDI) or Speckle-Tracking echocardiography (STE)^23,24^, both echocardiographic imaging techniques. In this study, the results from the optical image analysis were compared to results typically obtained by TDI. The TDI data used for comparison originates from the left ventricle, whereas the DIC data is obtained from the surface of the right ventricle, and therefore the absolute values are not fully comparable. It should be noted that conventionally when analyzing TDI data, the overall changes in shape are of higher interest than absolute values. All strain and deformation values, i.e. principal strains, vector components, etc. presented in this work were obtained from the same imaging sequence over five consecutive heartbeats. Strain and strain rate measurements have also been done from right ventricle and some recommendations of the usability have been made by European Association of Cardiovascular Imaging (EACVI) and The American Society of Echocardiography (ASE)^23,25,26^. The main principles of strain and strain rate measurements are the same for the right ventricle, but normal values and weight of abnormal values are not yet known well enough.

### Vector components

**Figure 4:**
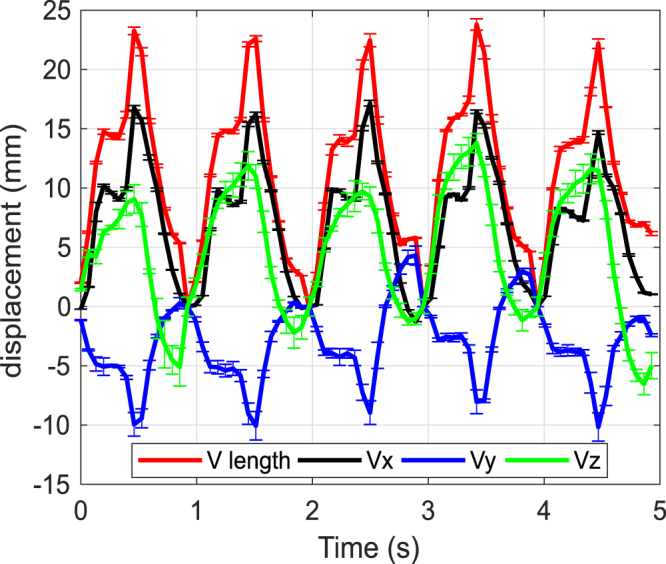
The length of the displacement vector and the components of the displacement vector obtained from the DIC analysis and the standard deviation error bars for each point.

Figure 4 shows the displacement in the X, Y, and Z-directions and the overall length of the displacement vector as a function of time obtained as an average of the vectors from the rectangular shaped area of interest, shown in Fig. 3, as well as the standard deviation error bars for each data point.

The shape of the pulses for the vector length (V_l_) and its X-component (V_x_) are more or less similar. During each cardiac cycle, both of them first increase to approximately two thirds of the maximum value, then decrease slightly before increasing to the peak value. From this point, the V_l_ and V_x_ drop at a high rate, reaching the minimum at the end of the pulse. The maximum values for the V_l_ and V_x_ are around 25 mm and 15 mm, respectively. V_y_ is also similarly shaped, reaching maximum values of around 10 mm in absolute movement. Note that by convention, the observed movement to the left during systole is showing negative values. Unlike the V_l_ and the V_x_ components, which return approximately back to the reference position at the end of each cardiac cycle, V_y_ exceeds to positive values at some points, more specifically during the third and fourth pulses, i.e., the surface moves to an end-diastole position further right when compared to the initial reference position.

In comparison, the Z-component (V_z_) goes through a slightly different behavior at each pulse. The V_z_ values increase continuously to the maximum of approximately 10 mm and then gradually decrease towards zero, mostly lacking the more distinct cardiac cycle characteristics expressed particularly in V_x_ and V_l_.

### Principal strains

**Figure 5:**
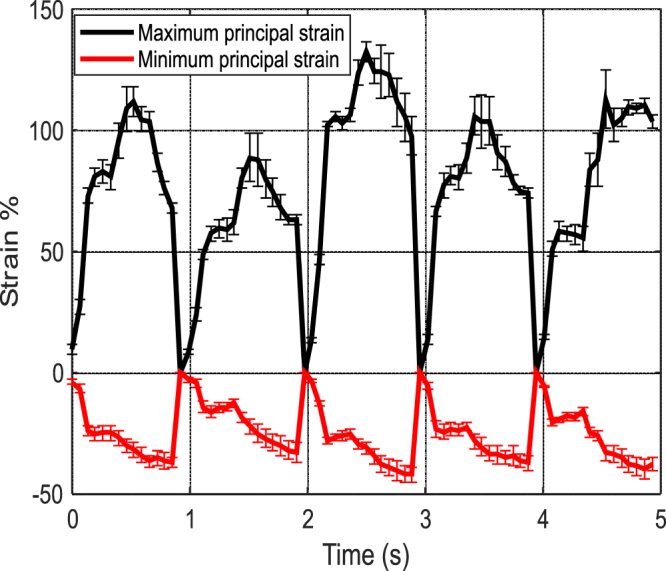
Average maximum and minimum principal strains obtained from the DIC analysis and the standard deviation error bars for each point.

Figure 5 shows the average maximum, minimum principal strains and the standard deviation error bars for each data point obtained from the area indicated by the black rectangle in Fig. 3. The maximum normal strains are positive and the highest values for each cycle are reached close to the middle of the cycle, giving each cycle a pulse-like shape. Conversely, the minimum normal strains are negative reaching the highest absolute values towards the end of each pulse. The maximum normal strains reach values between 100–150 percent, indicating very strong tensional deformation of the surface of the heart when using the end-systole stage, i.e., the cardiac cycle stage where the volume is at its minimum, as a reference state. The minimum principal strains reach values between −30 to −45 percent of deformation. The overall change in the pulse amplitudes for both principal strain values is behaving conjointly during the five heart beat cycles. For example, for both principal strains, the largest and lowest pulse amplitudes are at the second and third cardiac cycle, respectively.

### Tissue velocity

Tissue velocity describes the speed and direction at which the myocardium moves during the cardiac cycle and is typically used for assessment of the diastolic and systolic functions of the left ventricle. Tissue Doppler Imaging (TDI) uses the Doppler principles to measure the velocity of the myocardial motion (tissue velocity)^27^, utilizing the shift in the frequency of the ultrasound signals that are reflected from the moving parts of the heart. However, this technology has its own limitations as it can only measure the motion parallel to the direction of the ultrasound beam. Furthermore, the signal is subjected to high noise, making the interpretation of the data rather challenging and the measurements difficult to reproduce^28^.

**Figure 6:**
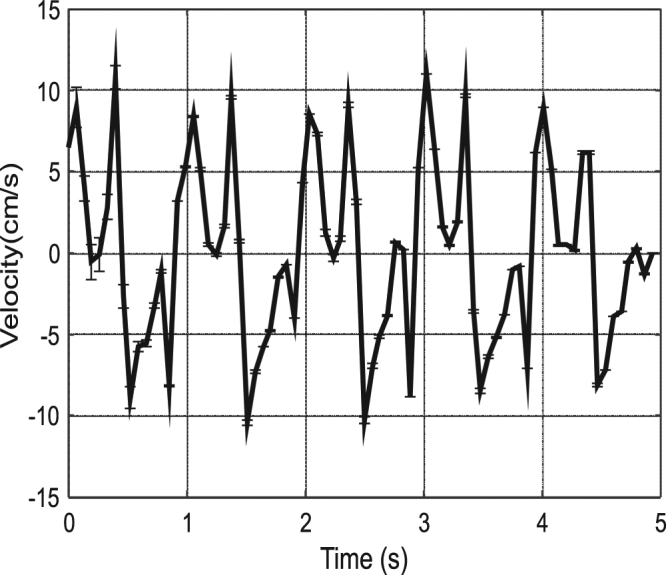
Tissue velocity values calculated from the vector length and interframe time and the standard deviation error bars for each point.

The tissue velocity can be calculated from the displacement data obtained at a known frequency by dividing the change in the length of the displacement vector by the interframe time. Figure 6 shows the average tissue velocity obtained from the black rectangle area depicted in Fig. 3. The tissue velocity varies between +10 and −10 cm/s, and each pulse reveals the same overall shape characteristics with minor scatter in the velocity values.

**Figure 7:**
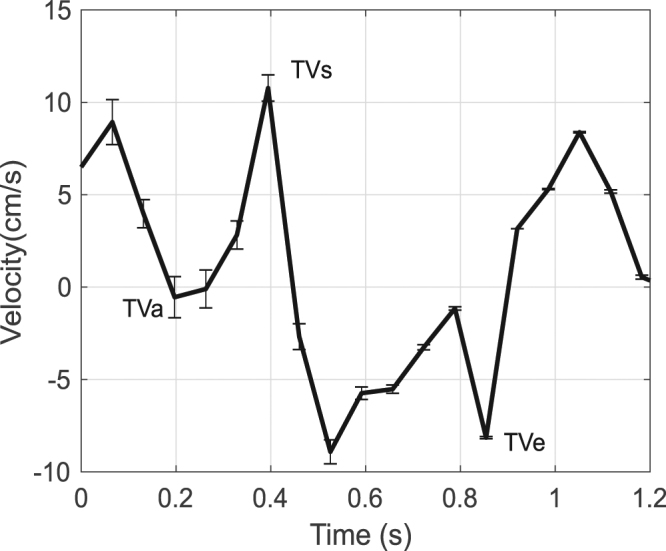
Tissue velocity calculated using DIC and the standard deviation error bars for each point.

Conventionally in the medical field, various characteristic points are observered in the tissue velocity vs. time graphs. The most useful or most important points include peak systolic tissue velocity (TV_s_), peak early diastolic tissue velocity (TV_e_), and peak late diastolic tissue velocity (TV_a_). Figure 7 shows the DIC measurements as a close-up of Fig. 6. The overall shape of the graph and the values are in good accordance with the tissue veclovity values determined by TDI found in literture and used as reference in the clinical routines^29^. There are various naming conventoions used in the medical field to denote the charactaristic points in the tissue velocity vs time graph, here we use the follwing where TV_e_ is the maximum value of velocity in the negative direction, which is by convention the movement away from the apex. A local maximum negative velocity value (TV_a_) is found between TV_e_ and the maximum positive (towards the apex) velocity value, denoted as TV_s_.

### DIC Fractional shortening

Fractional shortening (FS) is routinely used for the assessment of the ability of the heart to pump blood into the circulation. Fractional shortening is the relative change in the length of a selected dimension of the heart between the dilated and contracted states. As typically obtained by the ultrasound methods, fractional shortening describes the cross sectional shortening of the ventricle from standard projections. The exact location from where the fractional shortening is obtained depends on the projection of the ultrasound image and there is inter-observer and intra-observer variability in measurements. A comparable value can be obtained with DIC using a virtual extensometer as shown by the red line shown in Fig. 3. Although the mathematical definition of fractional shortening is the same for both methodologies, the values are obtained from different locations of the heart, and therefore the absolute values are expected to be different. With DIC, the fractional shortening for each heart beat is obtained by recording the length of a line between two fixed reference points for each image as a function of time. The length of the line for each image is normalized by the maximum length of the line, i.e., in reference to the point in time when the ventricle is in the maximum dilated state for each particular heartbeat (the end-diastolic state). Mathematically this can be expressed as

1 $${\boldsymbol{FS}}({\boldsymbol{t}})=(1-\frac{{\boldsymbol{L}}({\boldsymbol{t}})}{{{\boldsymbol{L}}}_{{\boldsymbol{\max }}}})\times 100 \% =\frac{{{\boldsymbol{L}}}_{{\boldsymbol{\max }}}-{\boldsymbol{L}}({\boldsymbol{t}})}{{{\boldsymbol{L}}}_{{\boldsymbol{\max }}}}\times 100 \% $$

where L(t) is the length of the virtual extensometer line as a function of time and L_max_ is the maximum length of the line for that particular heartbeat. In conventional heart monitoring with ultrasound technology, the fractional shortening is defined as

2 $${\boldsymbol{FS}}=(\frac{{\boldsymbol{LVEDD}}-{\boldsymbol{LVESD}}}{{\boldsymbol{LVEDD}}})\times 100 \% $$

where LVEDD and LVESD are the left ventricle end-diastolic and end-systolic dimensions, or dimensions corresponding to the maximum volume and minimum volume, respectively. Depending on the measurement method, normal lower limits of FS values describing left ventricular function are 25% for M-mode and 18% in the direct 2D mode measurements^23^.

**Figure 8:**
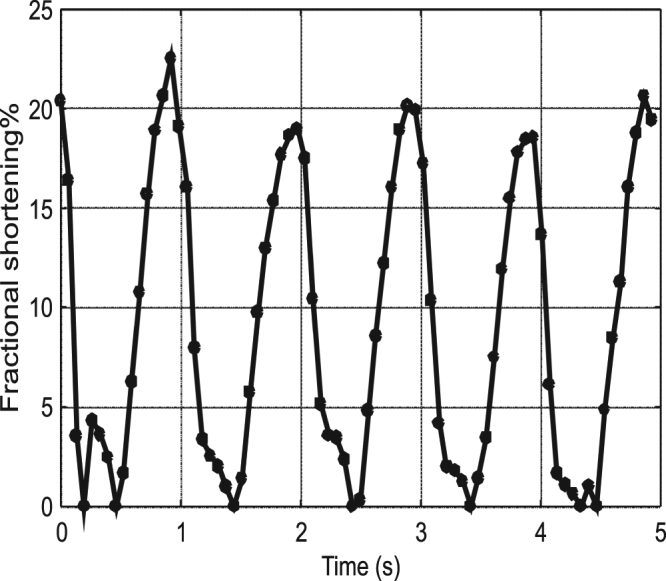
Fractional shortening obtained from the DIC analysis.

The fractional shortening obtained from the DIC analysis as a function of time is shown in Fig. 8. The level of fractional shortening is approximately constant over the duration of five heartbeats, showing maximum values of around 20%. At each cycle, the fractional shortening increases steadily to the maximum and goes through a plateau during the decrease to the minimum.

### Strain and strain rate

**Figure 9:**
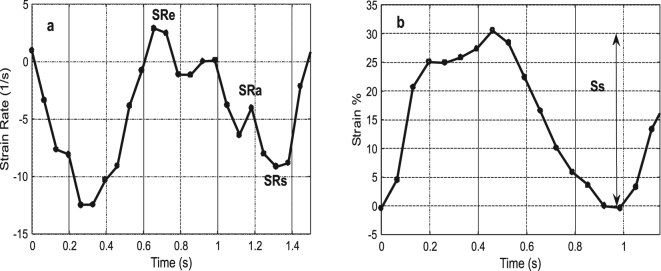
(**a**) Strain rate extracted by DIC b) Strain extracted by DIC.

Strain and strain rate are among the most recent and most promising indicators for the evaluation of myocardial functions^30^. Figure 9a shows the average engineering strain and strain rate as a function of time obtained from the DIC analysis (derived from the red line and black area marked in Fig. 3) for one heartbeat out of the recorded 11 heartbeats. The average peak strain for all 11 consecutive heartbeats is 33.25% with a standard deviation of 1.7%. The measurement is very stable and the results are repeatable. Like tissue velocity vs time discussed in the previous section, the strain and strain rate measurement results are similar to the results obtained by TDI^29^. In the strain rate as a function of time graph the important characteristic points comprise the peak systolic strain rate (SR_s_), peak early diastolic strain rate (SR_e_), and peak late diastolic strain rate (SR_a_). In Fig. 9a, the strain rate reaches a maximum at the start of the pulse (SR_e_), reveals a second but weaker maximum (SR_a_) after that before reaching the maximum negative value at the end of the cycle (SR_s_). Quite analogous to the tissue velocity data, the overall shape of the strain rate as a function of time plot and the changes in the curve shape are of higher interest than the absolute strain rate values from a clinical perspective, which can vary significantly from patient to patient. Taking a similar approach DIC recording of the strain data as a function of time is shown in Fig. 9b. The peak right ventricular strain (S_s_) is the difference between the maximum positive and negative strains for each cycle.

**Figure 10:**
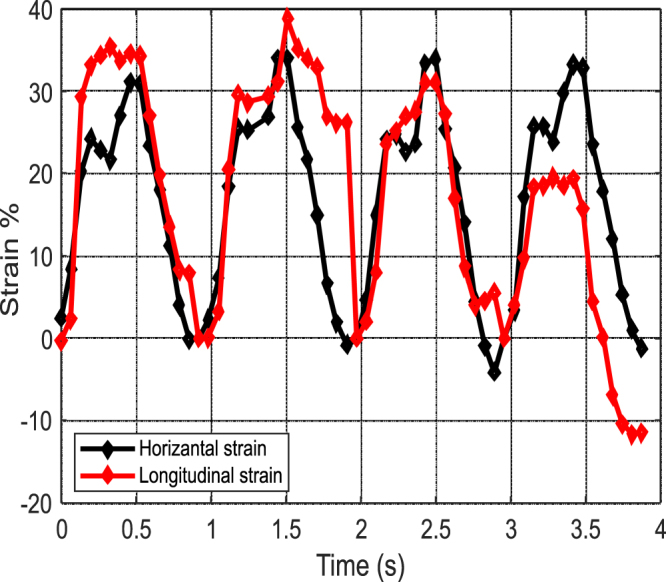
Horizontal and longitudinal strains as a function of time obtained by DIC.

Figure 10 shows horizontal and longitudinal strains for three heartbeats. The horizontal strain is measured on a direction perpendicular to the red line shown in Fig. 3. The measured strains show that the heart compresses essentially similarly in both directions for this particular patient.

## Discussion

DIC provides a contact free method for the quantitative evaluation of the biomechanical response and functioning of the heart. It can produce similar or comparable parameters as established ultrasound (u/s) technology, but also some unique parameters that cannot be obtained with current u/s methods. Although noticeable similarities can be seen in the strain, strain rate, and velocity plots extracted using TDI and DIC, various differences in both techniques need to be pointed out. The first major difference is the position from which the data is extracted. TDI relies on detecting the shift in frequency of the ultrasound signals that is reflected from moving objects. This information is then used to quantify the myocardial velocities from *inside* of the heart^29^. DIC, however, has only access to the images from the *outer surface* of the heart, and only a section of the heart is visible at any time. Consequently, DIC measures the displacement from the visible surface of the right ventricle and/or atrium. Furthermore, the physical principles underlying both techniques are distinctly different which has be considered when interpreting the results. While with TDI the measured frequency shift results in direct measurement of velocity and requires signal integration to obtain displacement, DIC measures displacements and velocity or strain rate are obtained by the time derivatives (at known frame rates). Accurate determination of time derivatives therefore requires use of higher acquisition frame rates.

The interventricular wall, or septum, generates a considerable proportion of the right ventricle ejection energy. Therefore, the obtained displacement and velocity data always includes the motion due to interaction with the left ventricle and the atria, as well as the interaction with the lungs. In other words, the motion of the surface of the right ventricle is a superimposition of “rigid body movement” (translational, rotational), and contractional activity of the ventricle. Additionally, the respiration of the patient during the surgery leads to slight modulation movement of the heart towards and away from the cameras. Although the stereo DIC system is not as sensitive to out of plane motion as a 2D DIC system, this might introduce slight errors in the generated results^31^. In fact, we have observed the movement of the heart due to respiration whenever several heartbeats were analyzed. In translation vector based time series data, the movement manifests itself in an amplitude modulation following the frequency of the respiration cycle.

All this can explain, at least partly, the observed differences between the strain, strain rate and tissue velocity measurements obtained by DIC and TDI. The DIC measurement of speed includes the motion caused by the left side of the heart as well, which pushes the right side away in the competition for space. An important point to mention is that the values discussed here can vary significantly from patient to patient^28,30,32^. The differences can be related to the patient’s health conditions, the geometrical projection and position constraints and conditions during TDI data acquisition, or simply the physiological variations in patient’s heart functions in general. For this reason, the medical relevance of the various measurements presented here should not focus primarily on absolute values, but on the shape of the plots and especially on the relative changes that occur in the overall behavior of the heart at different stages of the surgery or between different patients. However, this comparison requires more data and it will be the focus of future papers.

It is important to note that since the image is rotated by the software in order to keep the X and Y directions horizontal and vertical (during either calibration or defining new X and Y directions) it can be said that the X and Y-axis directions are user dependent. Consequently, V_x_ and V_y_ are dependent on the X and Y axes in relation to image rotation. Unlike V_x_ and V_y_, the vector length is independent of axis directions, making it a more suitable indicator of the heart deformation compared to the other vector components. In a clinical setting, user and experience independent descriptors of the function of the heart are more usable and easier to adopt. Therefore, orientation independent values (such as vector length and principal strains) show better potential to be adopted in the clinical routine.

### Limitations of the method

- In many cases, especially regarding the senior patients, the surface of the heart is covered with a layer of fat. Although the epicardial fat layer differs in its biomechanical properties from the myocardium, its thickness is only a few millimeters^33^ and it follows the movement of the below muscle tissue closely. This will limit the strain resolution of the measurement, but as the required resolution is rather low, it is safe to assume that the errors introduced by the epicardial fat layer are manageable. Most importantly, in the clinical use of the DIC the measurement would always be compared to the native stage of the same patient, so any errors due to the epicardial fat layer would not affect the comparison for the patient’s condition during the later stages of the surgery. Finally, it should be noted that typically in DIC measurements, the target surface is already covered with an extra layer for providing adequate contrast. This layer deforms with the underlying material and it is often differs vastly in properties with the material it covers.
- Since this measurement requires sternotomy, the comparison of the measurement results to a healthy subject is impossible. However, the true clinical and scientific value of a control results on a healthy heart is questionable. The pathophysiology of the heart surgery patients varies considerably and the comparison to a healthy heart would not give significant added value. The only feasible references for the DIC measurements are the results describing the functioning of the heart in the beginning of the surgery right after the sternotomy before any surgical repairs. This reference data can be used for comparison of the measurement results after the surgical repairs and/or after any medical interventions.
- The visible area of the heart that is accessible for the DIC measurement is only a portion of the full surface of the heart (Fig. 2), and therefore, the measured strains do not cover the whole ventricle. However, this limitation is not very significant for effective clinical use. Recent studies have shown^34–36^ that very localized strain measurements focusing only on the small region over the annulus, or the area where the tricuspid valve attaches to the myocardium, is enough to predict the ejection fraction of the right ventricle. The limited view is therefore enough to characterize the systolic function of the right ventricle. This is quite much similar to ultrasonic speckle tracking where the longitudinal strains are measured from several centimeters long segments of the heart depending on the overall size of the ventricle. Consequently, the section of the heart used for strain measurements in DIC is similar to these more stablished methods.

## Conclusion

A new methodology was described for obtaining data on the functioning of the heart using optical imaging. Some features of the biomechanical response of the heart were described using values typical for mechanics studies using digital image analyses, whereas some clinically relevant and established parameters were mimicked and compared to TDI measurements. The obtained full field measurements provided new insight into the biomechanics of the heart. The new methodology could be proven to be a promising tool for future studies, for instance, on the complete description of the effects of various pharmaceutical agents, e.g., inotropes, on the mechanical function, movement, and biomechanical response of the right ventricle. The main conclusions of the work can be summarized as:

- The strain, strain rate, and tissue velocity data obtained with DIC was found to be comparable to the data obtained with TDI. The characteristic values that are typically obtained from the TDI data could also be obtained from the DIC analyses.
- Additional data for describing the response of the heart can be obtained using DIC. This includes principal strains, length of the deformation vectors and the vector components. Since these values are commonly used for mechanical behavior analysis, future studies can focus on their relation with heart functions in conjunction with echocardiography/ultrasound data.
- Optical imaging and suitable image analysis algorithms have the potential to be used as an automated analysis and monitoring method, which do not overly rely on the user experience and capability. The flexible data acquisition, high spatial resolution, and better visualization of the functioning of the heart make optical image analysis a suitable candidate for further studies as a new method for patient monitoring during cardiac surgery.

### Further research topics

The results presented in this work demonstrate that Digital Image Correlation (DIC) can be used for monitoring and analyzing the functions, behavior, and biomechanical response of the right ventricle during open-heart surgery. This opens a variety of research areas to explore, such as the effects of respiration and positive end-expiratory pressure (PEEP) on the deformation and functioning of the RV. In addition, simultaneous and ECG synchronized DIC and TDI/Thallium Stress Test (TST) measurements should be carried out to establish a better correlation between the DIC and TDI data.
